# Surgical Cases Communicated to D. Gilbert, M. D., Professor of Surgery in the Medical Department of Pennsylvania College, Philadelphia

**Published:** 1852-04

**Authors:** 


*Surgical Cases communicated to* D. GILBERT, M. D., Professor of Surgery in the Medical Department of Pennsylvania College, Philadelphia.

To the Editors of the Medical Examiner.

The cases herewith sent I believe to be sufficiently interesting to secure for them a place in your valuable monthly periodical. The first two cases are from my friend, NATHANIEL WATSON, M. D., of Lancaster county, Pennsylvania.

CASE. I. In the summer of 1825, I was called to Jos. Bougher, at Graysbills mill. I found him, about an hour and a half after the accident, lying upon a mattrass on the floor. As I entered the room, Mrs. G. said, "Doctor, Joseph is dead." I discovered a dislocation of the cervical vertebræ below the origin of the phrenic nerve. I placed a foot on each shoulder, grasped the head firmly and stretched the neck with a rotary motion. The vertebræ returned to their place; the head had been twisted to one side. He began to breathe visibly very soon after. After rubbing the extremities for some time, his pulse returned—continuing the frictions with spirits, he opened his eyes in about an hour after the dislocation was reduced, and attempted to sit up. He became frantic in another hour, with hard and rapid pulse, for which he was bled freely. He was kept in bed, in a dark room, aperients were given, with cooling acidulated drinks and low diet, which was strictly enjoined for five days. On the eighth day he was able to walk through the room, free from all unpleasant symptoms and mind perfectly clear. In two weeks from the time of the accident he returned to work." He had sustained the injury by falling from a wagon loaded with hay, and was taken up for dead by the other hands.

Case II. "On the 14th of July, 1842, I visited a grandson of Esquire Missimores, who, 22 hours previously, had fallen from a cherry tree. The head was twisted to the left side, there was no feeling in the extremities, pulse feeble, respiration slow and stertorous, pupils dilated, extremities cold, &c. I pronounced it a case of dislocation of the vertebræ of the neck, and gave it as my opinion, that the boy would die if the luxation was not reduced; and, at the same time informed him, that he might die during the attempt, as well as subsequently to reduction. I reduced as in the case of Bougher. Frictions were made to the extremities, and spirits applied to them for one hour before he was was restored to consciousness, when he complained of pain in the neck and head. Respiration and pulse were gradually restored, and the extremities became warm. Ordered an enema, and requested that he should be kept quiet.

15th. Pain in back of head, pulse hard and quick, took ℥xij. of blood from the arm, applied cups to the temples, ordered purgative mixture, and then to apply a blister to the nape of the neck, and preserve quietness.

16th. Found him much better—continue quietude and spare diet.

18th. Continues to improve, and is apparently out of all danger.

22d. Discharged him."

These cases are exceedingly valuable in demonstrating that the vertebræ of the neck may be dislocated, and yet recovery follow. All the cases hitherto published, so far as my reading has extended, have had a fatal result. There are two cases reported in St. Bartholomew's Hospital reports, one of which died on the same day, the other in 32 hours after the accident. Another case is reported by Mr. Stanly, Royal Hospital, Harlan, died on 3d day. A case occurred in the New York Hospital, which terminated fatally on the 3d day ; and the last case recorded may be found in the Boston Medical and Surgical Journal for August last, furnished by E. K. Sandborn, M. D., of Lowell, Massachusetts, death on the 8th day.

CASE. III. Furnished by my friend J. G. Lightner, M. D., of Shirleysburg, Huntingdon county, Pennsylvania, presents points of interest, and therefore is also respectfully submitted for publication.

On the 15th of June, 1851, I was called to see a female child of Mr. B.'s, æt. four years, small of its age, and very thin in flesh. I found her with a slight fever and somewhat restless. On examination I discovered in the left lumbar region a tumor, which was moveable, and in size about that of an infant's head at birth. Immediately above the umbilicus there was a projection about two inches broad, and not more than three-fourths of an inch thick, as if some hard substance was pressing from within. Pressure made on it with the fingers did not produce pain or uneasiness. This was not moveable. I made up my mind that it was a tumor, but as to its nature I could not decide; and the part that projected above the umbilicus I thought might be some osseous substance, from its hardness. I gave the child a cathartic, which operated freely, and directed a febrifuge solution, and in a few days she was as well as usual. She never enjoyed good health after she was a year old, and her mother thought she felt a lump in her side long before she said anything about it. I did not see the child again until some time in October last, but occasionally heard that the lump was on the increase, that the hard projection had descended below the umbilicus, and finally was lost in the tumefaction of the abdomen in general. During this time her parents had given her various vermifuges, from the belief that it might be worms, and indeed some of the neighbors had persuaded them that it was a large tape worm.

Dr. Brewster and I went out some time in the above month to see the child, and we were not a little surprised to find its abdomen extended from above the scrobiculus cordis to the pubes, and laterally to fully the size of a woman's abdomen at her full period of gestation. The lower extremities were œdematous and very much swollen, and her upper extremities, together with her face, were very much emaciated. On percussion of the abdomen

I found no fluctuation; the abdomen had the same dull sound, in all its parts, indicating solidity. We concluded that the operation of paracentesis would not increase the danger, for the case was hopeless. I selected about midway between the umbilicus and pubes; after dividing the cutis, I inserted the trochar about two inches, and found considerable resistance; I withdrew the stilet, and nothing followed but blood, which amounted to about half an ounce; the child was somewhat relieved for a few days, but her abdomen continued to increase in size, if possible, so that the cutis seemed as if it would give way and let out the whole contents. She became more restless, œdema of the lower extremities and emaciation of the upper extremities increased, and on the 20th November at two o'clock, A. M., she died. I had previously obtained leave of the parents to make a post obit examination. Dr. Brewster being from home, I had no medical assistance. I made the examination of the case about 4 o'clock, P. M., (assisted by two women.) On opening the abdomen by an incision from the scrobiculus cordis to the pubes, the tumor made its appearance, which I found, by introducing my hand, had not formed any very extensive adhesions; I could easily separate them with my hand as I passed it around the tumor, but there was some difficulty in doing this for want of room. I discovered that its pedicle was about four inches in extent. I divided it with a scalpel from its attachment, and found it to be pretty solid, spherical in form, and composed of two lobes. I believe now that it was the corner or edge of one of these lobes that formed the solid projection above the umbilicus. Its weight is seventeen pounds and a quarter, and measures thirteen inches in diameter. It appears to be an adipose tumor, with a cavity of small extent filled with bloody serum, and a substance resembling soft cheese. The intestines were crowded to the right side, and had a natural appearance; the liver was forced upwards against the diaphragm; the kidney was rather higher than usual, and the aorta and ascending vena cava passed through under the tumor; these vessels appeared to suffer from pressure, and I believe that the tumor was nourished from a branch of the aorta, which was sent off a little distance above its bifurcation.

